# More Distally Located Duodenal Webs: A Case Series

**DOI:** 10.21699/jns.v5i4.435

**Published:** 2016-10-10

**Authors:** Rahul Gupta, Praveen Mathur, Sharanabasappa Gubbi, Pradeep Kumar Gupta, Ramendra Shukla, Anu Bhandari

**Affiliations:** 1 Department of Paediatric Surgery, SMS Medical College Jaipur, Rajasthan, India-302004; 2 Department of Radiodiagnosis, SMS Medical College Jaipur, Rajasthan, India-302004

**Keywords:** Duodeno-jejunal junction, GERD, Unusual locations, Duodenal web

## Abstract

Duodenal atresia is a frequent cause of intestinal obstruction in the newborn. Obstruction due to duodenal web is infrequent, but its location other than second part of duodenum is rare with only a few cases reported in the literature. We are reporting three patients where we found duodenal webs at unusual locations. In one neonate the web was located at third part of duodenum and in other two patients the web was present at duodeno-jejunal junction (DJ).

## INTRODUCTION

Duodenal atresia (DA) is a common cause of neonatal intestinal obstruction. [1] Obstruction due to duodenal web (type I) is infrequent. [2] The second part of duodenum is commonly involved, while involvement other than second part of duodenum is rare. [2] We came across three patients with duodenal web at unusual location. We propose that, failure of recanalization shifted from the site of "embryological traffic jam" to unusual locations (third part of the duodenum and DJ junction).

## CASE SERIES

**Case 1:**

A 3-day-old, preterm female neonate, weighing 1.3 kg, born by vaginal delivery was presented to our department with bilious vomiting, failure to pass meconium and epigastric fullness since birth. Antenatal ultrasounds were not done. On examination, the neonate was hemodynamically stable. The abdomen was soft and non-distended. Laboratory investigations were normal. An abdominal X-ray showed gastric and duodenal gas (C-shape) shadow extending beyond the second part of duodenum along with absence of distal air (Fig.1A); Laparotomy revealed dilated stomach and duodenum and increased wall thickness till DJ junction correlating with the X-ray findings (Fig.2). Medial visceral rotation and kocherisation of the duodenum was performed to completely expose the DJ junction. Second part of the duodenum was normal. After palpation of a membranous structure in the lumen in relation to palpable ring extra-luminally at DJ junction, an enterotomy was performed by a longitudinal incision (on the anti-mesenteric border). It revealed a mucosal diaphragmatic web (Fig.2). Luminal patency of the distal bowel was confirmed with normal saline. Excision of web was performed circumferentially. Postoperatively patient had continued downhill course, developed sepsis and died on 3rd postoperative day. 

**Figure:**
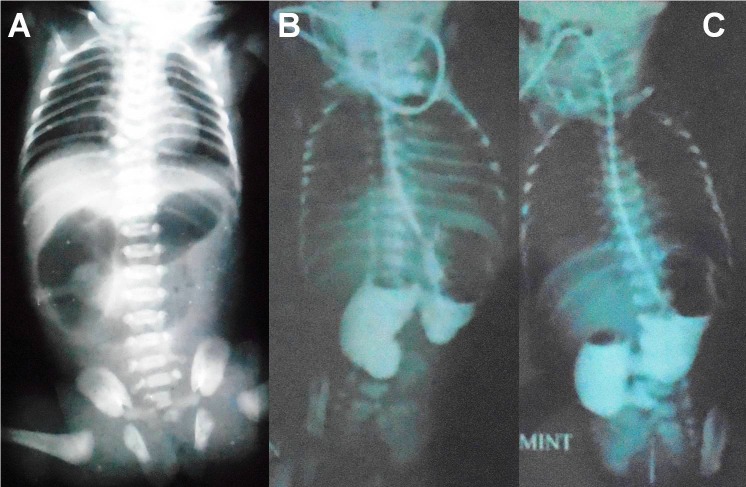
Figure 1: Abdominal X-ray showing gastric and duodenal gas (C-shape) shadow extending beyond the second part of duodenum along with absence of distal air (A); upper gastro-intestinal contrast study suggesting obstruction at the third part of the duodenum (B,C).

**Figure:**
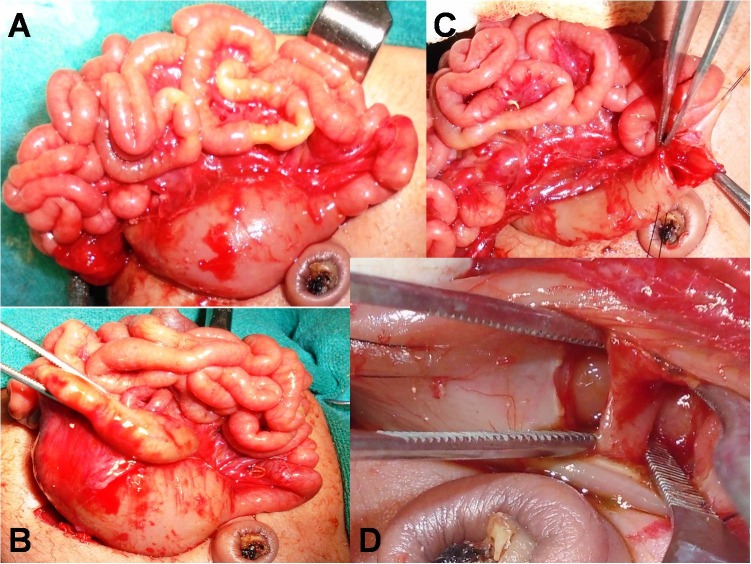
Figure 2: Intra-operative images depicts dilated duodenum and increased wall thickness till DJ junction (transition zone) with complete exposure of the DJ junction (A,B); enterotomy at DJ junction revealing web (C,D).

**Case 2:**

A 2-day-old, preterm female neonate, weighing 1.9 kg, 1st in birth order was born by vaginal delivery. He presented to our institute with bilious vomiting; there was no delayed passage of meconium. On examination, child was hemodynamically stable, anicteric, mildly dehydrated. Abdominal signs included mild epigastric distension, soft on palpation and absent bowel sounds; nasogastric aspirate was bilious. Laboratory tests were normal. Abdominal ultrasonography (USG) was not conclusive. Upper gastro-intestinal contrast study was performed which suggested duodenal obstruction (Fig.1B). Laparotomy revealed dilated stomach and duodenum till third part, with a change in the caliber beyond it (Fig.3). C-loop of the duodenum was exposed. Palpation of palpable fibrotic ring extra-luminally at third part of duodenum was done. Enterotomy and circumferential excision of web (membrane) at the third part of duodenum was carried out (Fig.3). The postoperative course was uneventful and the child is doing well at follow-up.

**Figure:**
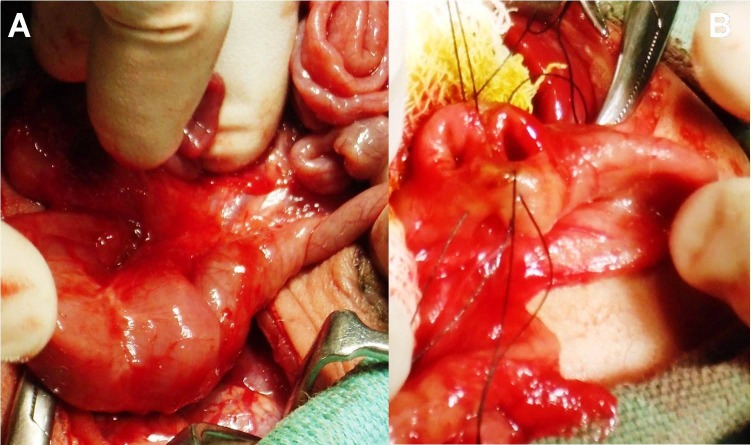
Figure 3: Intra-operative images showing dilated duodenum till third part (fibrotic ring extra-luminally) of duodenum with a change in the caliber beyond it (A); Enterotomy and circumferential excision of web (membrane) at the third part of duodenum completed.

**Case 3:**

A 5-month-male infant presented to us with bilious vomiting for the last few days. He had episodes of non-bilious vomiting, minutes after breast feeding since birth, which was being managed medically. The symptoms aggravated only when weaning was started few days back. Child also had dry cough and fever for the last few days. Medical records revealed that patient was given treated for gastroesophageal reflux disease (GERD) since birth. On examination, the patient was stable with mild dehydration, respiratory rate-38/min, and pulse rate-110/min, with weight-5.5 kg. On physical examination, epigastric distention was present, fine crepitations were heard on chest auscultation. Laboratory investigations revealed TLC-12,400 mm3, hemoglobin-9 gm%, SGOT-109 IU/L, and SGPT-90 IU/L. Abdominal X-ray showed a dilated stomach; chest radiograph suggested pneumonitis. Patient was stabilized and treated for pneumonitis. Ultrasonography was suggestive of gastro-duodenal dilatation. An upper gastrointestinal (UGI) contrast study demonstrated gastroesophageal reflux, distended stomach and markedly dilated duodenum till the DJ junction with normal distal bowel pattern, suggestive of incomplete intestinal obstruction (Fig.4). Obstruction due to the web was suspected with high index of suspicion for windsock anomaly. This precipitated laparotomy which revealed markedly dilated stomach and duodenum and increased wall thickness till DJ junction (Fig.5). At DJ junction, an enterotomy was performed by a longitudinal incision (on the anti-mesenteric border) which revealed a mucosal diaphragmatic web (Fig.5). Web was having an eccentric opening (perforation). Excision of web was performed circumferentially. Patient is healthy at 6 months follow up. 

**Figure:**
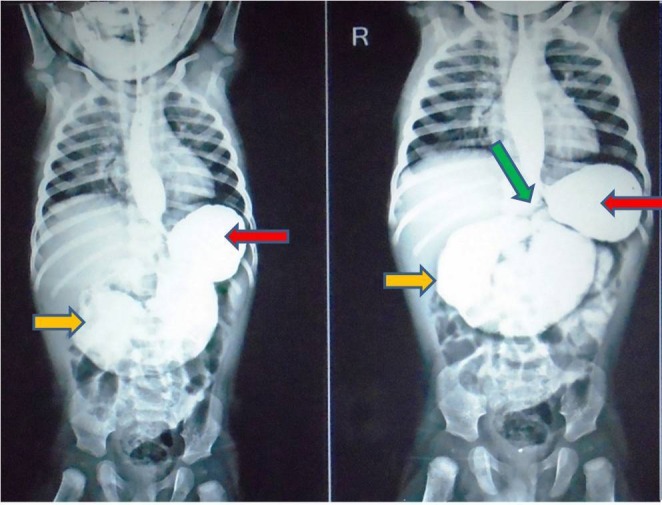
Figure 4: Upper gastrointestinal contrast study (the images taken at 5 and 15 minutes) showing gastroesophageal reflux, dilated stomach (red arrow) and markedly distended duodenum (orange arrow) up to DJ junction (green arrow). Caliber of jejunum and rest of the distal bowel appears normal.

**Figure:**
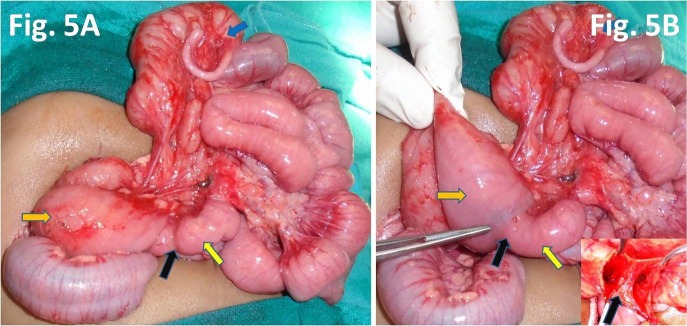
Figure 5: Intra-operative images depicts markedly dilated duodenum with increased wall thickness (orange arrow) till transition zone (black arrow) at DJ junction, and jejunum (yellow arrow) appears normal (Fig.5A); marked disparity in the size of the duodenum (orange arrow) and jejunum (yellow arrow). Inset image reveals web (black arrow) at DJ junction (Fig.5B).

## DISCUSSION

Bilious vomiting is the main presenting symptom and plain radiography (showing double bubble appearance) is the most valuable diagnostic tool in all cases of DA except those with partial or incomplete obstruction and also in patients with web at unusual location (additional bubble/gas shadow is seen) as seen in two of our neonates. [1-5] In case of incomplete obstruction, presentation may mimic gastroesophageal reflux disease (GERD), making the diagnosis difficult and delayed for months as seen in case 3. [5,6] Therefore a high index of suspicion for incomplete obstruction is required in a child presenting with repeated episodes of vomiting since birth. Other symptoms include dehydration, upper abdominal distension, poor weight gain and failure to thrive. Conditions like midgut volvulus, annular pancreas, duplication cysts, superior mesenteric artery syndrome, and preduodenal portal vein are differential diagnoses which also need surgical intervention.[1-7] Screening with ultrasonography is of paramount importance. An UGI contrast study is indicated to demonstrate the site and nature of the obstruction in case of incomplete obstruction and also help in surgical planning. 

Usually all types of DA are limited to the second (post-ampullary) followed by first part of the duodenum. The second part of the duodenum is also the most common site of web (85%); involvement other than second part of duodenum is rare. [1,5] Incomplete obstruction may be caused by a web with a central or eccentric opening as seen in case 3. Rarely, the web may be stretched distally because of peristalsis and high intraluminal pressure and is known as 'windsock anomaly'. [6] Kaddah et al reported patients with type-I DA; the mucosal diaphragmatic web was present at the second part of the duodenum, except three patients with webs at unusual location. [7] Caution should be exercised as the windsock deformity may arise from 2nd part of duodenum but may cause dilatation till the DJ junction. Therefore before opening the duodenum the surgeon should be sure of the origin of the web, as suggested by palpable fibrotic ring extra-luminally, as seen in our cases. Complete excision of web is important as it may regrow in case of incomplete excision and lead to recurrent obstruction. [6] 

Boyden et al. described the second part of duodenum as the "embryological traffic jam". [9] Epithelial proliferation followed by occlusion (5th to 6th week) and failure of recanalization and re-vacuolization is the proposed embryogenesis of duodenal atresia; with second part of duodenum recanalizing at the end. [10] Boyden also suggested the relation (as both conditions arise from second part of duodenum) between duodenal atresia and associated biliary ductal anomaly. [9] Also, two cases of gallbladder duplication associated with duodenal atresia have been reported [8,11] In all three cases in this series, biliary ductal and gall bladder anomalies were not seen and second part of duodenum was normal. We propose that, failure of recanalization shifted from the site of "embryological traffic jam" to unusual locations (third part of the duodenum and DJ junction) conspicuous by the absence of biliary ductal and gall bladder anomalies in our cases.

In conclusion, duodenal web may present at location other than second part of duodenum, as seen in three of our patients with web at third part of duodenum and DJ junction. Clinicians should have high index of suspicion for webs in a child presenting with vomiting since birth.

## Footnotes

**Source of Support:** Nil

**Conflict of Interest:** None
